# Long-term changes in subjective daytime sleepiness following tonsillectomy and uvulopalatopharyngoplasty (UPPP): A prospective cohort study

**DOI:** 10.1097/MD.0000000000050856

**Published:** 2026-10-23

**Authors:** Przemysław Bant, Aleksander Julian Jurkiewicz, Kornel Szczygielski, Julia Łukawska, Michał Kaczmarczyk, Dariusz Jurkiewicz

**Affiliations:** a Department of Otolaryngology and Oncological Laryngology with Cranio-Maxillofacial Surgery Unit, Military Institute of Medicine: National Research Institute, Warsaw, Poland.

**Keywords:** Epworth Sleepiness Scale, sleep disorders, subjective daytime sleepiness, tonsillectomy, UPPP

## Abstract

Diseases of the palatine tonsils may be associated with varying degrees of subjective daytime sleepiness (SDS), a symptom that may affect daily functioning. This study evaluated long-term changes in SDS in patients before and after tonsillectomy or uvulopalatopharyngoplasty (UPPP). The primary aim of this study was to evaluate long-term changes in SDS following tonsillectomy or UPPP. Secondary objectives included the assessment of patient satisfaction and the influence of demographic and clinical factors, including sex, age, chronic diseases, tonsil size, and histopathological findings, on treatment outcomes. A prospective cohort study included 105 adult patients, with an 85% follow-up rate: 89 patients (26 women, 63 men). SDS was assessed using the Epworth Sleepiness Scale before surgery and after a mean follow-up of 6 years. Additional analyses considered demographic and clinical variables. Patient satisfaction was measured using a visual analog scale and willingness to undergo repeat surgery. Both tonsillectomy and UPPP were associated with a significant long-term reduction in SDS. The mean ESS score decreased from 7.29 preoperatively to 4.37 at follow-up (*P* < .05). A clinically meaningful improvement in ESS of ≥2 points was achieved in 71.9% of patients, and the proportion of patients with normal ESS scores (≤10) increased from 75.3% to 92.1% (*P* < .001). While the unadjusted ESS reduction was greater after UPPP than after tonsillectomy (4.22 vs 1.81 points), multivariable analysis demonstrated a significant interaction between surgical procedure and baseline ESS, indicating that the magnitude of improvement differed between procedures according to baseline subjective sleepiness. Sex, tonsil size, histopathological findings, and chronic diseases did not significantly influence the outcomes. Tonsillectomy and UPPP were associated with a sustained, long-term reduction in SDS. The magnitude of postoperative improvement increased with baseline subjective sleepiness, with the relative difference between procedures becoming greater at higher baseline ESS values. These findings suggest that upper airway surgery may be associated with durable improvements in patient-reported daytime sleepiness, including among patients with baseline ESS scores within the non-EDS range.

## 1. Introduction

Subjective daytime sleepiness (SDS) refers to an individual’s perceived tendency to fall asleep or difficulty maintaining wakefulness during intended waking hours.^[1–3]^ Clinically significant excessive daytime sleepiness (EDS) represents the more severe end of this spectrum and is associated with neurobehavioral deficits, reduced quality of life, and increased safety risks.^[4–10]^ However, SDS exists on a broader continuum, and changes in perceived sleepiness may affect general well-being and daily functioning even when scores do not reach the clinical threshold commonly used to indicate EDS.

Surgical interventions of the upper airway, such as tonsillectomy and uvulopalatopharyngoplasty (UPPP), are performed for various clinical indications, including recurrent tonsillitis, upper airway obstruction, and socially disruptive snoring.^[11]^ Although these procedures have been shown to improve daytime sleepiness in patients with overt sleep-disordered breathing,^[12–17]^ their effects across the broader spectrum of SDS in clinical cohorts remain less well characterized. Furthermore, despite the growing body of evidence regarding upper airway surgery, prospective data on long-term patient-reported outcomes remain limited, and direct long-term comparisons of tonsillectomy and UPPP with respect to SDS are scarce.

Therefore, the primary aim of this study was to evaluate long-term changes in SDS following tonsillectomy or UPPP. The secondary aim was to assess whether changes in SDS differed according to histopathological diagnosis and to examine their associations with sex, age, comorbidities, tonsil size (Pirquet scale), visual analog scale (VAS) scores, and willingness to undergo repeat surgery.

## 2. Methods

A prospective cohort study was conducted including 105 patients undergoing tonsillectomy or UPPP at the Department of Otolaryngology and Head and Neck Oncology, Military Institute of Medicine - National Research Institute in Warsaw. The study was designed as an observational cohort study comprising 2 groups of patients undergoing different surgical interventions (tonsillectomy vs UPPP), without the inclusion of a nonsurgical control group. The study protocol was approved by the institutional ethics committee, and written informed consent was obtained from all participants. The patient cohort included 72 men (68.6%) and 33 women (31.4%). The mean age was 42 years for men and 35 years for women. Six years after surgery, we were able to follow-up with 89 patients (85% of those initially qualified for surgery), including 26 women (29%) and 63 men (71%). Patients who underwent surgical treatment were examined in person by one of the authors of the study, which eliminated discrepancies in the preoperative assessment.

All patients underwent a detailed medical history assessment and physical examination. The otolaryngological evaluation included, among other elements, an assessment of palatine tonsil size using the Pirquet scale.^[18]^

Inclusion criteria were as follows:

- adults (≥18 years);
- palatine tonsillitis (PT) diagnosed based on medical history and physical examination, according to SIGN criteria, with at least 5 episodes per year for a minimum of one year.
- tonsillar hypertrophy diagnosed based on clinical assessment, including patients with snoring and/or obstructive sleep apnea syndrome (OSAS).
- history of peritonsillar abscess.

Exclusion criteria included:

- diagnosed malignancy of the palatine tonsils.
- history of palatine tonsil biopsy.
- confirmed or clinically suspected immunodeficiency.

Detailed clinical classification and qualification criteria have been described in our previous study.^[18]^ OSAS status was based on previously available clinical documentation and was not systematically reassessed using a standardized sleep study across the entire cohort. Therefore, objective measures of OSAS presence or severity were not available for all patients and were not included as variables in the present analysis.

Tonsillectomy was performed in 48 patients (54%) and UPPP in 41 patients (46%). Significant baseline differences were observed in age (40.48 ± 11.33 vs 51.00 ± 12.43 years) and body mass index (BMI) (27.55 ± 5.98 vs 29.98 ± 4.73 kg/m^2^) between groups (*P* < .05 for both). Given baseline differences between groups, comparisons should be interpreted with caution. In all patients, the palatine tonsils were removed and subsequently subjected to histopathological evaluation. Based on the histopathological findings, 2 diagnostic groups were identified: patients with palatine tonsil hypertrophy (PTH) and patients with PT. Group allocation was based on immunohistochemical analyses, as previously described.^[18]^ The mean age at the time of surgery was 36.1 years (range: 21–70 years; median: 35) in the tonsillectomy group and 46.3 years (range: 28–70 years; median: 44) in the UPPP group. Most participants (53%) were young adults (≤44 years), whereas middle-aged adults (45–64 years) and older adults (≥65 years) accounted for 36% and 11%, respectively. Age classification was based on definitions provided by the United States Census Bureau.^[19]^

SDS was assessed using the Epworth Sleepiness Scale (ESS). The ESS is a validated Polish-language tool that is simple and widely used by clinicians and researchers to assess daytime sleepiness.^[2,20,21]^ The scale was developed in 1991 based on data obtained from both healthy individuals and patients with various sleep disorders. Its purpose is to evaluate the overall level of daytime sleepiness, as opposed to transient feelings of fatigue occurring at a specific moment. The ESS is a self-administered questionnaire consisting of 8 items, each rated on a 4-point scale (0–3), designed to assess the likelihood of dozing off or falling asleep during 8 typical daily activities. The total score is the sum of all items and ranges from 0 to 24, with higher scores indicating greater SDS.^[22]^ According to standard interpretive criteria, scores of 0 to 10 are considered normal, 11 to 12 indicate mild EDS, 13 to 15 moderate, and 16 to 24 severe EDS.^[23]^ For the purposes of the present analysis, ESS scores of 0 to 10 were considered to fall within the non-EDS range, whereas scores >10 were considered indicative of EDS.^[24,25]^ Based on the findings of Patel et al the minimal clinically important improvement on the ESS ranges from −2 to −3 points.^[26]^ However, a meta-analysis by Crook et al indicates that a 2-point change may already be clinically meaningful.^[27]^

During the preoperative medical interview, the presence of comorbidities was recorded, with particular attention to cardiovascular, metabolic, endocrine, respiratory, gastrointestinal, genitourinary, and renal diseases, as well as autoimmune and rheumatologic disorders, musculoskeletal and neuromuscular conditions, and hematologic and psychiatric disorders. The study also assessed whether patients, based on their experience with the surgical treatment, would choose to undergo the same procedure again. Patients were asked whether, knowing both the benefits of the treatment and its associated inconveniences: such as postoperative pain, hospitalization, and preoperative stress: they would consent to the same surgery again.^[28]^ In addition, patients’ overall satisfaction related to the surgical procedure was evaluated using a VAS ranging from 0 to 10 points. Assessments were made by patients after completing the surgical treatment, taking into account their entire experience with the procedure.^[28]^ During the follow-up period, no additional treatments affecting sleep-disordered breathing (such as continuous positive airway pressure therapy, further surgical interventions, or structured weight-loss programs) were introduced.

Descriptive statistics were calculated for continuous variables as mean ± standard deviation and median with interquartile range (IQR), and for categorical variables as counts and percentages. The normality of distribution was assessed using the Shapiro–Wilk test. Parametric tests (paired and independent *t* tests) were applied for normally distributed data, while nonparametric tests (Wilcoxon signed-rank test and Mann–Whitney *U* test) were used when the assumption of normality was not met. Effect sizes (*r* for Wilcoxon test and mean differences with 95% confidence intervals [CI] for *t* tests) were calculated to assess the magnitude of observed effects. The magnitude of change in ESS scores between independent groups (e.g., sex, age categories, type of surgery) was evaluated using the independent *t* test for normally distributed data and the Mann–Whitney *U* test for non-normally distributed data. For comparisons across more than 2 independent groups (e.g., age subgroups), the Kruskal–Wallis test was applied, followed by post hoc pairwise comparisons with Bonferroni correction when significant differences were detected. Categorical ESS outcomes (<11 points vs ≥11 points) before and after surgery were compared using the McNemar test.

Because the surgical groups differed significantly in age and BMI, and because baseline ESS may influence the magnitude of postoperative improvement, an adjusted multivariable linear regression analysis was performed to evaluate the association between surgical procedure and postoperative ESS. Postoperative ESS was used as the dependent variable, while surgical procedure (tonsillectomy vs UPPP), preoperative ESS, age, and BMI were included as independent variables. To assess whether the association between surgical procedure and postoperative ESS differed according to baseline daytime sleepiness, an interaction term between surgical procedure and preoperative ESS was included in the model. The final model was specified as:


postoperative ESS~surgical procedure×preoperative ESS+age+BMI.

Because the interaction between surgical procedure and preoperative ESS was statistically significant, adjusted postoperative ESS and adjusted changes in ESS were estimated at selected baseline ESS values (5, 10, and 15 points) using estimated marginal means. Pairwise comparisons between surgical groups were performed at each baseline ESS value. For the adjusted analyses, age and BMI were treated as continuous variables, while surgical procedure was treated as a categorical variable, with tonsillectomy used as the reference category. Statistical significance was set at *P* < .05. A post hoc power analysis based on the observed effect size (Cohen’s *d* = 0.72) and sample size (n = 89) demonstrated excellent statistical power (>99%) to detect differences between paired measurements. This cohort study was reported in accordance with the Strengthening the Reporting of Observational Studies in Epidemiology guidelines. All analyses were performed using R software (R Foundation for Statistical Computing, Vienna, Austria).

## 3. Results

We successfully contacted 85% of all patients initially enrolled in the study and subsequently treated surgically. The remaining participants were unreachable, declined participation, or returned incomplete questionnaires; one patient died due to causes unrelated to the intervention. The severity of daytime sleepiness, assessed using ESS, was evaluated before surgery and at a 6-year follow-up. The mean ESS score in the entire cohort decreased from 7.29 ± 4.34 preoperatively to 4.37 ± 3.58 at the 6-year follow-up. The median ESS also decreased from 7 (IQR 4–10) to 4 (IQR 1–6). This reduction was statistically significant (Wilcoxon signed-rank test: V = 2853.5, *P* < .05) with a large effect size (*R* = 0.767) (Fig. 1). To assess clinical relevance, we calculated the proportion of patients achieving the (minimal clinically important difference defined as a change of ≥2 points). Overall, 71.9% of patients met or exceeded this threshold, indicating that the majority experienced a clinically meaningful reduction in daytime sleepiness.

**Figure 1.**
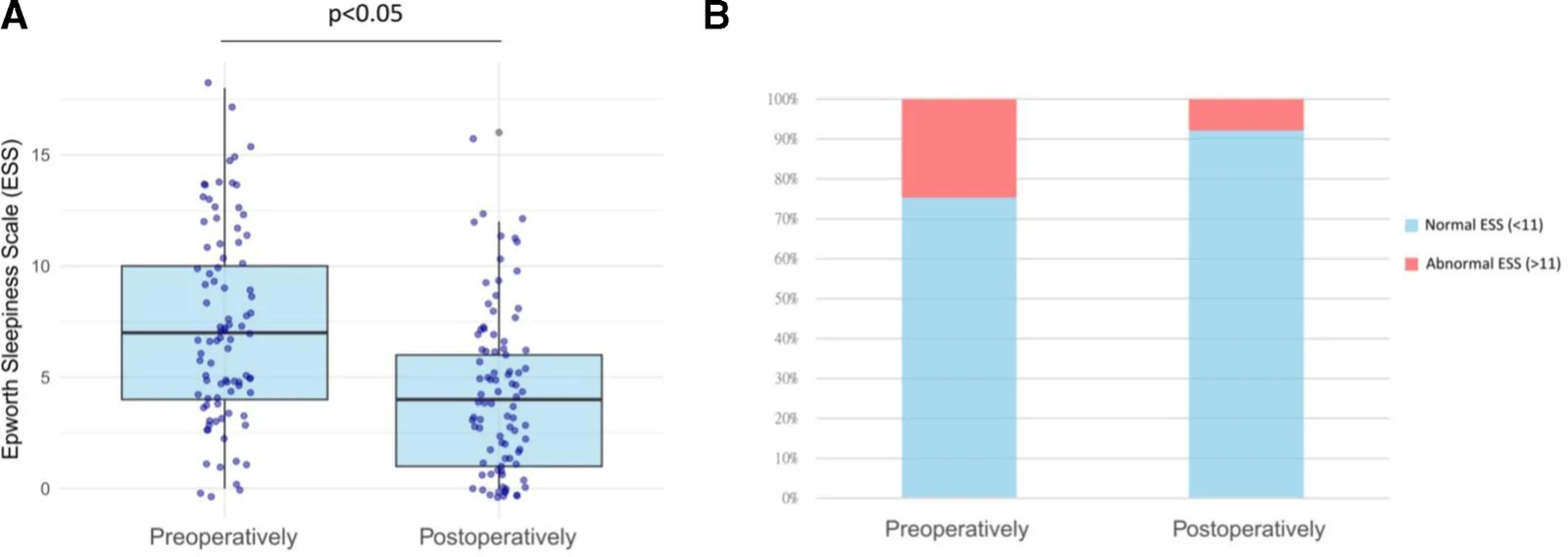
A) Distribution of pre- and postoperative ESS scores; B) Proportion of patients with normal ESS scores (<11) before and after surgery. ESS = Epworth Sleepiness Scale.

### 3.1. Analysis by type of surgery

The analysis included patients who underwent tonsillectomy (n = 48) and UPPP (n = 41). Both groups showed a statistically significant reduction in daytime sleepiness following surgery. In the tonsillectomy group, the mean ESS score decreased from 7.21 ± 4.44 preoperatively to 5.40 ± 3.81 postoperatively. The mean reduction was 1.81 points (95% CI: 1.36 to 2.26), which was statistically significant (paired *t* test: t(47) = 8.09, *P* < .05). In the UPPP group, the mean ESS score decreased from 7.39 ± 4.27 to 3.17 ± 2.90. The mean reduction was 4.22 points (95% CI: 3.01–5.43), also reaching statistical significance (paired *t* test: *t*(40) = 7.05, *P* < .05).

In the unadjusted analysis, the magnitude of ESS improvement was greater in the UPPP group than in the tonsillectomy group (mean reduction: 4.22 ± 3.83 vs 1.81 ± 1.55 points, respectively). The between-group difference in change scores was 2.41 points (95% CI: 1.12–3.69; independent *t* test: *t*(51) = −3.77, *P* < .001). However, the surgical groups differed significantly in age and BMI, and therefore the unadjusted comparison of change scores was further evaluated using a multivariable linear regression model.

The model demonstrated a significant interaction between surgical procedure and baseline ESS (*β* = −0.477, SE = 0.099, *P* < .001), indicating that the magnitude of improvement differed between procedures.

according to baseline ESS. Adjusted estimates showed an increasing difference in ESS improvement between the 2 procedures with increasing baseline ESS. At baseline ESS values of 5, 10, and 15 points, the adjusted improvement was 2.52, 5.88, and 9.23 points after UPPP, compared with 1.46, 2.43, and 3.40 points after tonsillectomy, respectively. The corresponding adjusted differences were 1.06 (95% CI: 0.02–2.10), 3.45 (95% CI: 2.37–4.52), and 5.83 (95% CI: 4.04–7.62) points, respectively (Table 1).

**Table 1:** Adjusted differences in ESS improvement between UPPP and tonsillectomy according to baseline ESS.

Baseline ESS	Tonsillectomy	UPPP	Difference (UPPP−tonsillectomy)	*P*-value
5	1.46 (0.80–2.13)	2.52 (1.78–3.27)	1.06 (0.02–2.10)	.045
10	2.43 (1.73–3.14)	5.88 (5.11–6.64)	3.45 (2.37–4.52)	<.001
15	3.40 (2.21–4.59)	9.23 (7.93–10.54)	5.83 (4.04–7.62)	<.001

Values are adjusted mean improvements in ESS; values in parentheses represent 95% confidence intervals. Positive differences indicate greater improvement following UPPP. Estimates were adjusted for age, BMI, and baseline ESS, including the surgical procedure × baseline ESS interaction.

ESS = Epworth Sleepiness Scale, UPPP = uvulopalatopharyngoplasty.

### 3.2. Analysis by sex

In the male group (n = 63), the mean ESS score decreased from 7.41 ± 4.20 (IQR 4.0–10.5) preoperatively to 4.14 ± 3.31 (IQR 1.0–6.0) postoperatively, representing a statistically significant improvement (Wilcoxon signed-rank test: *V* = 1495, *P* < .05) with a large effect size (*R* = 0.769). Among females (n = 26), a reduction in daytime sleepiness was also observed, with the mean ESS score decreasing from 7.00 ± 4.74 to 4.92 ± 4.18. The mean reduction was 2.08 points (95% CI: 1.39–2.76), also reaching statistical significance (paired *t* test: *t*(25) = 6.25, *P* < .05). The mean reduction in ESS was greater in men than in women (3.27 ± 3.43 vs 2.08 ± 1.70 points, respectively), corresponding to a mean between-group difference of 1.19 points. The independent-samples *t* test indicated a statistically significant difference between sexes (*t*(84) = 2.19, *P* = .031). However, this difference was not confirmed by the Mann–Whitney *U* test (U = 952.5, *P* = .224). Because the distribution of ESS change scores was non-normal in men (Shapiro–Wilk test: W = 0.908, *P* < .001), the nonparametric comparison was considered more appropriate. Given the discrepancy between the parametric and nonparametric results, as well as the unequal sample sizes between men and women, the presence of a sex-related difference in the magnitude of ESS improvement remains uncertain.

### 3.3. Analysis by age

A statistically significant reduction in ESS scores was observed across all age groups following surgery. In young adults (19–44 years), the median ESS decreased from 6.89 (IQR 4.0–9.0) to 4.79 (IQR 1.0–6.5) (Wilcoxon signed-rank test: *V* = 683.5; *P* < .05, *R* = 0.736). In middle-aged adults (45–64 years), the median ESS decreased from 8.62 (IQR 5.0–12.25) to 4.53 (IQR 2.0–7.0) (*V* = 484.0; *P* < .05, *R* = 0.819), while in older adults (>64 years), the mean ESS decreased from 4.90 ± 3.00 to 1.90 ± 1.97 (paired *t* test: *t*(9) = 3.14, *P* < .05), with a mean difference of 3.00 (95% CI: 0.84–5.16). The greatest mean improvement was observed in middle-aged adults (4.09 ± 3.80), followed by older adults (3.00 ± 3.02), and the smallest in young adults (2.11 ± 2.21). Differences among groups were statistically significant (Kruskal–Wallis test: *χ*^2^ = 6.19; df = 2; *P* < .05, *R* = 0.27), with post hoc analysis showing a significantly greater improvement in middle-aged adults compared to young adults (*P* < .05). No significant differences were found between the other group pairs.

When patients were divided into 2 age categories (<45 years (n = 47) vs ≥45 years (n = 42)), both groups still showed significant reductions in ESS scores (<45 years: median pre 7.0, IQR 4.0–9.0, median post 5.0, IQR 1.0–6.5; *V* = 683.5; *P* < .05, *R* = 0.736; ≥45 years: median pre 7.0, IQR 4.25–11.75, median post 3.0, IQR 1.25–6.0; V = 762; *P* < .05, *R* = 0.803). However, younger patients, under 45 years of age, exhibited significantly smaller improvements (median change 2.0, IQR 0.0–3.0) compared with those ≥45 years (median change 3.0, IQR 2.0–6.0) based on the Mann–Whitney *U* test (*U* = 708; *P* < .05) with a moderate effect size (*R* = 0.250).

### 3.4. Analysis by histopathological classification

Patients were divided into 2 groups based on postoperative histopathological examination of the palatine tonsils: PTH and PT. Both groups showed a statistically significant reduction in daytime sleepiness after surgery. In the PTH group (n = 45), the mean ESS decreased from 8.24 ± 4.53 (IQR: 5.0–11.0) preoperatively to 4.89 ± 3.87 (IQR: 2.0–7.0) postoperatively (Wilcoxon signed-rank test: *V* = 768; *P* < .05, *R* = 0.79). In the PT group (n = 44), the mean ESS decreased from 6.32 ± 3.95 (IQR: 3.75–8.0) to 3.84 ± 3.21 (IQR: 1.0–6.0), also reaching statistical significance (Wilcoxon signed-rank test: *V* = 674; *P* < .05, *R* = 0.74). Comparison of the magnitude of ESS improvement between the 2 groups did not reveal statistically significant differences (Mann–Whitney *U* test: *U* = 1148; *P* = .19).

### 3.5. Analysis by patient’s readiness to undergo surgery again

Most patients (n = 79) reported that they would undergo the surgery again. In this group, the mean ESS decreased from 7.22 ± 4.33 (IQR: 4.0–10.0) preoperatively to 4.46 ± 3.63 (IQR: 1.5–7.0) postoperatively, and the reduction was statistically significant (Wilcoxon signed-rank test: *V* = 2279; *P* < .05, *R* = 0.765). Among patients who indicated they would not undergo surgery again (n = 10), a significant reduction in daytime sleepiness was also observed, with the mean ESS decreasing from 7.90 ± 4.63 to 3.70 ± 3.23 (paired *t* test: *t*(9) = 3.16; *P* < .05) with a mean difference of 4.2 (95% CI: 1.19–7.21). Comparison of the magnitude of ESS improvement between the 2 groups did not show statistically significant differences (Mann–Whitney *U* test: *U* = 316; *P* = .305).

### 3.6. Analysis according to the presence of chronic diseases

In the group of patients without any reported chronic diseases (n = 43), the mean ESS score decreased from 7.37 ± 3.89 (IQR: 4.0–10.0) preoperatively to 4.88 ± 3.56 (IQR: 2.0–7.0) at follow-up, representing a statistically significant improvement (Wilcoxon signed-rank test: *V* = 688; *P* < .05, *R* = 0.779). Similarly, in patients reporting chronic diseases (n = 46), a significant reduction in daytime sleepiness was observed, with ESS decreasing from 7.22 ± 4.76 (IQR: 3.25–10.75) to 3.89 ± 3.57 (IQR: 1.0–6.0) (Wilcoxon signed-rank test: *V* = 757; *P* < .05, *R* = 0.757). Comparison of the magnitude of ESS improvement between patients with and without chronic diseases did not reveal statistically significant differences (Mann–Whitney *U* test: *U* = 899; *P* = .457).

### 3.7. Analysis by tonsil size (Pirquet scale)

No statistically significant relationship was observed between the degree of PTH on the Pirquet scale and ESS scores.

### 3.8. Subjective well-being assessment (VAS)

No statistically significant association was observed between subjective well-being assessed on the VAS scale and ESS scores.

### 3.9. BMI correlation

There was no significant correlation between BMI and the change in ESS scores. Both Pearson (*R* = 0.19, 95% CI: −0.02–0.38, *P* = .077) and Spearman (*ρ *= 0.14, *P* = .179) analyses indicated a weak, non-significant association.

### 3.10. Categorized ESS scores

An analysis was performed using ESS score categorization, with values <11 points considered normal and ≥11 points indicating EDS. Before surgery, 67 patients (75.3%) had normal ESS scores, whereas at long-term follow-up, this number increased to 82 patients (92.1%). McNemar’s test demonstrated a significant change in category distribution after surgery (*χ^2^* = 11.529; *P* < .001), indicating a statistically significant shift in ESS category distribution toward the non-EDS range. Sixteen patients moved from the EDS to the non-EDS range; 72 *patients remained in the same category, and one patient showed deterioration.*

## 4. Discussion

Reduction of daytime sleepiness is one of the key indicators of the effectiveness of sleep disorder therapy.^[8]^ In the present long-term observational study, we demonstrated a statistically significant reduction in SDS following surgical treatment, including tonsillectomy and UPPP. The mean ESS score in the entire cohort decreased from 7.29 to 4.37 (Fig. 1A), and categorical analysis further showed a significant shift in scores toward normal values (<11 points), with the proportion of patients achieving normal ESS increasing from 75.3% to 92.1% (Fig. 1B), indicating the durability of the clinical effect. Despite the relatively low baseline severity of subjective sleepiness, which limited the potential for absolute score reduction, the observed improvement was both statistically and clinically meaningful. These findings support the potential for sustained, long-term improvements in patient-reported daytime sleepiness following both UPPP and tonsillectomy. Similar observations were reported by Lundkvist et al., who demonstrated a significant reduction in median ESS from 12 to 6 after UPPP (*P* < .001).^[29]^ Senchak et al also reported a significant decrease in SDS after isolated tonsillectomy in adults, with median ESS dropping from 12 preoperatively to 9 postoperatively (*P* = .014).^[15]^ Yaremchuk et al found a reduction in mean ESS from 11.1 to 5.5, corresponding to a 5.6-point improvement (*P* < .001).^[30]^ These findings support the effectiveness of UPPP and tonsillectomy in reducing SDS. Importantly, a substantial proportion of patients (71.9%) achieved a clinically meaningful improvement in ESS (≥2 points), further supporting the relevance of the observed changes despite relatively low baseline scores. However, unlike the studies mentioned above, which had relatively short follow-up periods, our analysis included a six-year follow-up, allowing assessment of the long-term sustainability of the treatment effect.

Reduction in SDS was observed across all analyzed subgroups, including those defined by sex, age, the presence of chronic diseases, or histopathological diagnosis of the excised palatine tonsils. The lack of a clear effect of sex on the magnitude of improvement aligns with most available publications, in which sex was not a significant predictor of treatment outcome.^[30,31]^ It should be noted, however, that some previous studies did not perform sex-specific analyses, likely due to underrepresentation of women in their cohorts.^[30,31]^ In our study, women accounted for 29% of participants, allowing sex-specific analyses, although the female subgroup was considerably smaller than the male subgroup. Although a greater mean reduction in ESS was observed in men than in women, this difference was not consistently demonstrated across statistical approaches. The independent-samples *t* test indicated a significant between-sex difference, whereas the Mann–Whitney *U* test did not. Given the non-normal distribution of ESS change scores and the unequal sample sizes between men and women (63 vs 26), the role of sex in determining the magnitude of postoperative improvement remains uncertain.

Age was associated with the magnitude of ESS reduction in unadjusted analyses, with the greatest improvements observed in middle-aged patients and those ≥45 years old (Fig. 2). This phenomenon may be explained by higher baseline sleepiness severity in these groups, providing a greater potential for postoperative improvement. Smaller reductions in younger patients were likely due to lower baseline ESS scores and, therefore, less room for improvement, limiting the possible range of improvement. The change in ESS was primarily driven by baseline scores, indicating that age was not an independent predictor of improvement magnitude. These findings are consistent with Sundman et al.,^[32]^ who found no independent effect of age on treatment outcome in multivariate analysis. Similar conclusions have been reported in other studies on the surgical treatment of obstructive sleep apnea, which found no significant association between age and SDS improvement.^[30,33,34]^ Therefore, the observed age-related differences mainly reflect baseline symptom severity rather than an independent effect of age.

**Figure 2.**
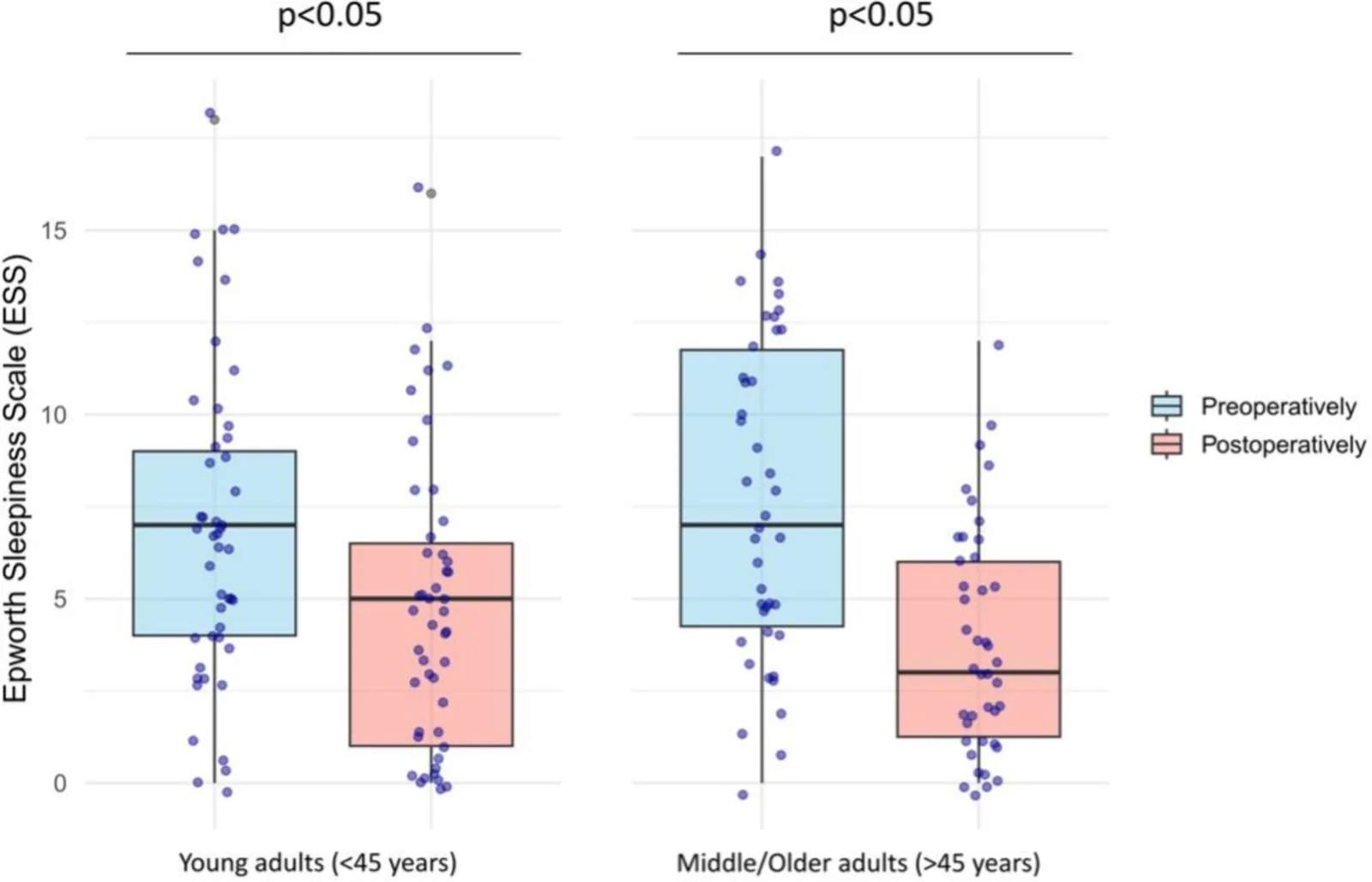
Comparison of the distribution of pre- and postoperative ESS scores across different age categories. ESS = Epworth Sleepiness Scale.

Significant differences in ESS improvement were observed between the 2 surgical procedures in the unadjusted analysis, with greater mean reductions following UPPP than tonsillectomy (Fig. 3). However, the surgical groups differed significantly in baseline age and BMI, and therefore the unadjusted between-group comparison should be interpreted with caution. In the adjusted multivariable analysis, the interaction between surgical procedure and baseline ESS was statistically significant, indicating that the difference in postoperative improvement between the procedures depended on the preoperative level of daytime sleepiness (Fig. 4). The adjusted difference in ESS improvement increased with increasing baseline ESS, from 1.06 points at a baseline ESS of 5 to 3.45 points at 10 and 5.83 points at 15 (Table 1). Thus, rather than indicating a uniform treatment effect of UPPP, our findings suggest that the relative difference between procedures may be more pronounced among patients with greater preoperative daytime sleepiness. Nevertheless, tonsillectomy alone was also associated with a clinically meaningful long-term reduction in ESS.

**Figure 3.**
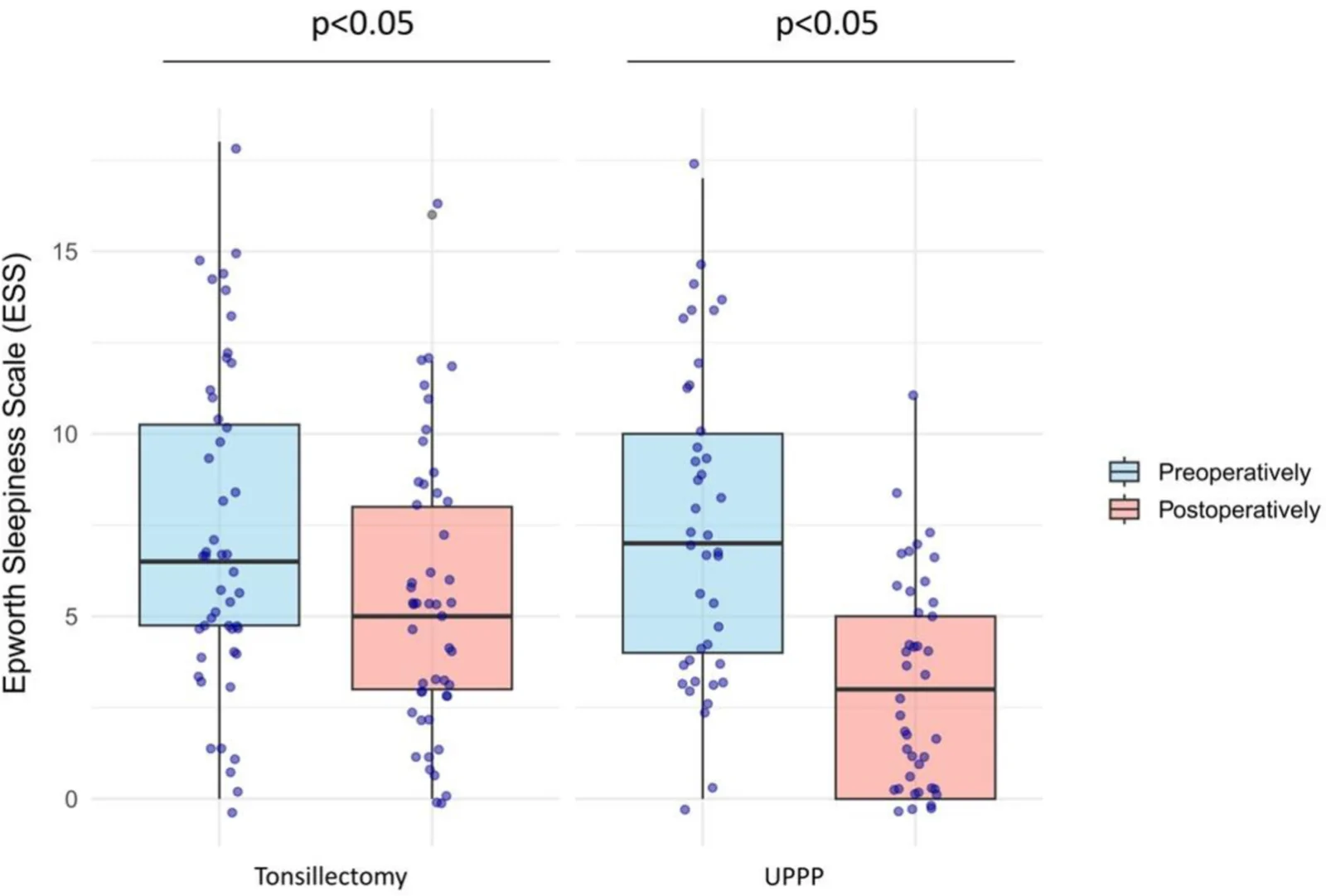
Distribution of preoperative and postoperative ESS scores according to surgical procedure. ESS = Epworth Sleepiness Scale.

**Figure 4.**
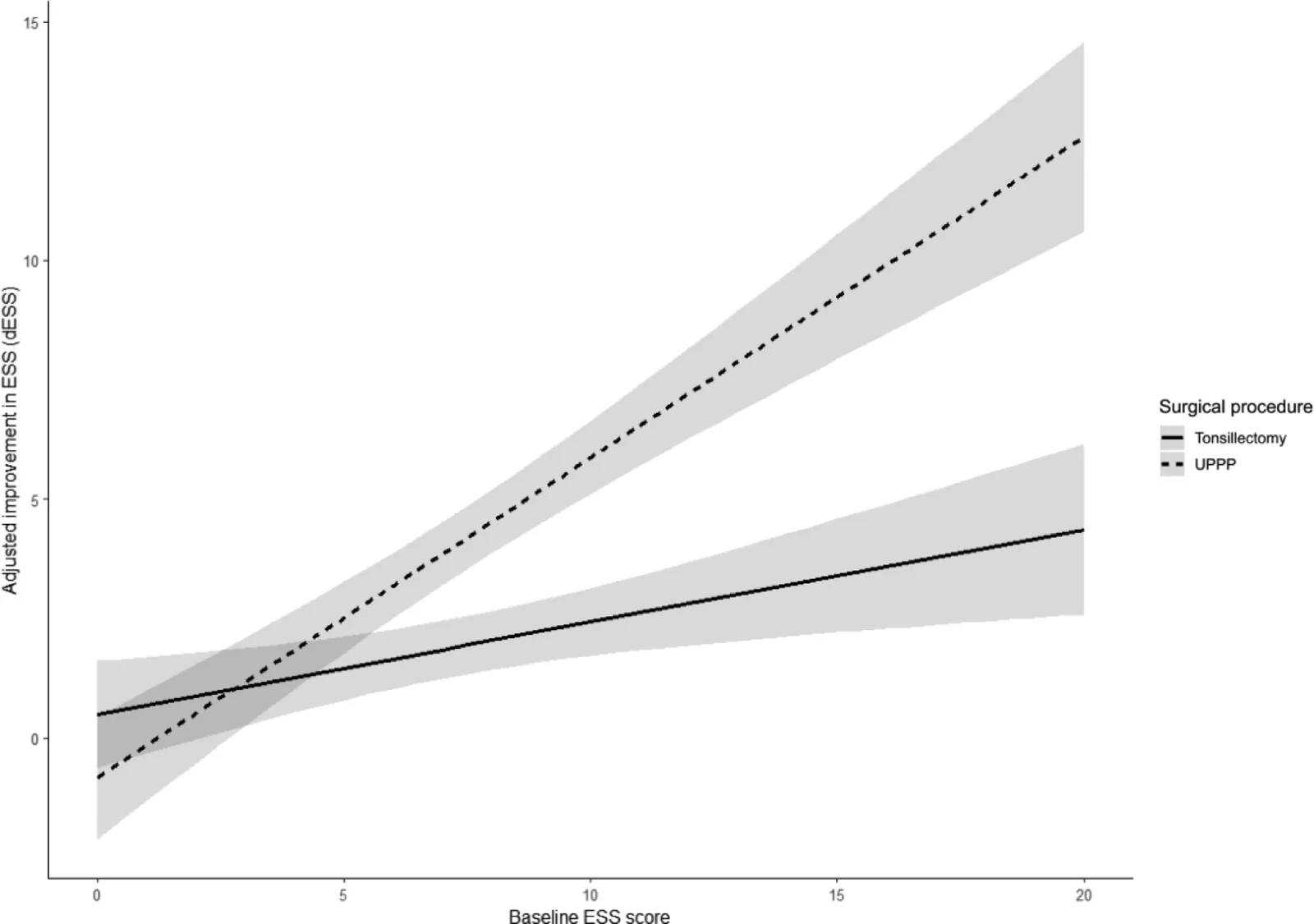
Adjusted change in ESS according to baseline ESS and surgical procedure. Shaded areas represent 95% confidence intervals. ESS = Epworth Sleepiness Scale.

These findings differ from those of the TEAMUP randomized trial, which reported no significant difference in ESS reduction between modified UPPP and isolated tonsillectomy.^[29]^ Several factors may account for this discrepancy. First, our study evaluated classical UPPP, involving more extensive tissue resection, whereas the TEAMUP trial used a modified UPPP technique. Second, differences in patient selection and baseline characteristics may have contributed to the different results. Importantly, the observational design of our study and the baseline differences between surgical groups preclude a direct causal comparison of the 2 procedures.

One might assume that larger tonsils, potentially causing greater upper airway resistance, would result in more pronounced improvement after removal. However, we found no statistically significant relationship between tonsil size on the Pirquet scale and the degree of SDS improvement. Nakata et al reported a significant SDS reduction after tonsillectomy but did not analyze the association between tonsil size and SDS improvement.^[35]^ The potential influence of tonsil size on SDS severity and response to surgery remains insufficiently understood and warrants further investigation.

To date, no studies have examined the relationship between histopathological diagnosis of excised tonsils and SDS reduction after surgery. In our analysis, surgical efficacy in reducing daytime sleepiness was comparable between PTH and PT groups, despite differences in baseline SDS severity. The absence of significant differences in ESS reduction indicates that the histopathological type of tonsil disease did not influence the clinical outcome, which could be a basis for further research on the pathophysiology of SDS.

Interestingly, we observed no significant effect of chronic diseases on ESS reduction after surgery. Although absolute improvement was numerically larger in patients with comorbidities, there were no statistically significant differences between subgroups, indicating comparable surgical efficacy regardless of baseline disease burden. This is clinically relevant because patients with elevated sleepiness often have multimorbidity.^[1]^

High VAS scores reported by patients after tonsillectomy and UPPP (8.57 and 8.18, respectively) indicate high satisfaction and confirm the long-term improvement in quality of life after surgery. The VAS captures the overall subjective assessment of treatment course and outcome, making it an appropriate tool for evaluating patient satisfaction and intervention effectiveness from the patient’s perspective.^[34]^ The willingness to undergo surgery again, previously unreported in the literature and analyzed in our prior study, is also relevant in assessing the value of surgical interventions. In our cohort, 88.8% of patients would choose to undergo surgery again, considering both benefits and complications. This is particularly notable as the decision reflects prior patient experiences, including postoperative pain, hospitalization, preoperative stress, and other inconveniences.^[28]^ High satisfaction has also been reported in other studies. Pinczel et al documented patient-reported improvement in both objective and subjective sleep parameters, despite incomplete cure, with high satisfaction rates.^[36]^ Similarly, Browaldh et al reported high satisfaction after surgery and few patients regretting the procedure.^[37]^ Conversely, Hicklin et al reported relatively low satisfaction after UPPP (mean 5.2/10), with 61% of patients indicating they would not undergo the procedure again.^[20]^ Importantly, low satisfaction was not solely related to lack of clinical efficacy; some patients with substantial clinical improvement still reported low satisfaction or unwillingness to repeat surgery.

Despite baseline differences in BMI between groups, no significant association was found between BMI and changes in ESS, suggesting that BMI did not meaningfully influence the observed outcomes. Nevertheless, baseline differences between groups, particularly in BMI and age, should be considered when interpreting the results.

This study has several limitations. The observational, non-randomized design introduces a risk of selection bias and limits causal inference. Baseline differences between groups, particularly in age and BMI, may have influenced comparative outcomes. Furthermore, without a nonsurgical control group, we cannot entirely exclude the influence of natural changes occurring over the approximately 6-year follow-up period, including aging, changes in body weight, lifestyle, and sleep habits, on the observed changes in SDS. In addition, the lack of assessment across the entire cohort prevented correlation of changes in SDS with objective measures of sleep-disordered breathing and precluded stratification of the observed changes according to the presence or severity of OSAS. The use of patient-reported outcomes also introduces a potential risk of recall bias. However, the study has important strengths. It is based on a prospective cohort with a high follow-up rate and provides long-term data with a 6-year observation period, for which long-term data remain limited. The use of clinically relevant endpoints, including the ESS and minimal clinically important difference, enhances the practical significance of the findings. These features strengthen the reliability of the findings and their clinical relevance.

## 5. Conclusions

Tonsillectomy and UPP were associated with sustained long-term reductions in SDS over a 6-year follow-up. The magnitude of improvement increased with baseline SDS, with the adjusted difference between procedures becoming greater at higher baseline ESS values. These findings suggest that upper airway surgery may improve patient-reported daytime sleepiness even among patients whose baseline ESS scores fall within the non-EDS range. Further controlled studies with standardized assessment of sleep-disordered breathing and OSAS severity are warranted to clarify the comparative effects of different surgical procedures.

## Author contributions

**Data curation:** Przemysław Bant, Aleksander Julian Jurkiewicz.

**Methodology:** Aleksander Julian Jurkiewicz, Przemysław Bant.

**Resources:** Przemysław Bant, Aleksander Julian Jurkiewicz.

**Validation:** Przemysław Bant, Kornel Szczygielski, Dariusz Jurkiewicz.

**Formal analysis:** Aleksander Julian Jurkiewicz, Julia Łukawska, Michał Kaczmarczyk.

**Investigation:** Aleksander Julian Jurkiewicz.

**Software:** Aleksander Julian Jurkiewicz, Michał Kaczmarczyk.

**Visualization:** Aleksander Julian Jurkiewicz, Julia Łukawska.

**Conceptualization:** Kornel Szczygielski, Dariusz Jurkiewicz.

**Supervision:** Kornel Szczygielski.

**Writing – original draft:** Aleksander Julian Jurkiewicz.

**Writing – review & editing:** Przemysław Bant, Kornel Szczygielski, Dariusz Jurkiewicz.
